# 肺癌干细胞研究进展

**DOI:** 10.3779/j.issn.1009-3419.2010.03.12

**Published:** 2010-03-20

**Affiliations:** 110001 沈阳，中国医科大学基础医学院病理教研室，中国医科大学附属第一医院病理科 Department of Pathology, College of Basic Medical Sciences, the First Affiliated Hospital of China Medical University, Shenyang 110001, China

肺癌是当今世界上对人类健康与生命危害最大的恶性肿瘤之一，肺癌的发病率和死亡率近年来呈明显上升趋势，严重威胁着人类健康。在我国的许多省市和地区，肺癌的发病与死亡近十年来一直居恶性肿瘤之首位。虽然临床上已经联合应用手术、化疗、放疗等治疗方案，但肺癌患者五年生存率仍不高，寻找新的治疗途径可能有利于肺癌的预后。

依据肿瘤干细胞理论推断，肺癌可能是一种干细胞疾病：具有自我更新和无限增殖能力的肺癌干细胞是肺癌发生的根源；肺癌的复发、侵袭转移、耐药、抗辐射等恶性表型特征都与肺癌干细胞有关。本文综合了肺癌干细胞最新研究进展及其在肺癌形成中的重要作用，同时为肺癌治疗提供了新的思路。

## 1 肺癌干细胞的发现与分离

### 1.1 利用细胞表面的分子标记分选肺癌干细胞

Kim等^[1]^利用流式细胞仪从大鼠细支气管和肺泡管结合部（the bronchioalveolar duct junction, BADJ）分离出一群CD45^+^/Pecam^-^/Sca-1^+^/CD34^+^细胞，其在正常情况下处于静止状态，当体内支气管和肺泡损伤时发生增殖，能够分化为其他类型的肺上皮细胞，表现出明显的自我更新特性，Kim将其命名为支气管肺泡干细胞（bronchoalveolar stem cells, BASCs）。体外培养中发现*K-ras*基因（原癌基因）激活可导致BASCs增殖更新；成鼠体内试验发现BASCs在肺癌发生早期可出现数目增多和体积增大，与肺癌起始有关，被认为是肺腺癌的靶细胞。最近，Regala等^[2]^发现非典型蛋白激酶C（protein kinase C, PKC）的激活可促进BASCs增殖和向肺癌干细胞的转化。

Eramo等^[3]^则认为肺癌干细胞属于CD133^+^细胞，然而上述类型肺癌干细胞的致瘤性较弱，在NOD^-^SCID免疫缺陷小鼠皮下需植入1×10^4^个CD133^+^细胞才能形成肿瘤，这些肺癌干细胞是否具有小鼠BASCs特征，目前尚不清楚。但最近有学者对此提出异议^[4]^，他们应用免疫磁珠从A549和H446肺癌细胞系中分离出CD133^+^细胞和CD133^-^细胞，二者均具备相似的自我更新、侵袭及耐受化疗药物能力。此外，40%以上的CD133^+^细胞和CD133^-^细胞均可形成较大克隆，这提示仅表达CD133尚不能作为A549和H446细胞系干细胞的标记物。Hayashi等^[5]^检测了CD133在小细胞肺癌（small cell lung cancer, SCLC）和非小细胞肺癌（non-small cell lung cancer, NSCLC）细胞系内表达，发现CD133几乎仅表达于小细胞肺癌细胞系，在非小细胞肺癌细胞系内很少表达，且CD133^+^小细胞肺癌细胞致瘤能力均强于非小细胞肺癌，但由于CD133表达过于广泛，不应作为分选肺癌干细胞的标记物。

最近Chen等^[6]^从肺癌标本及肺癌细胞系中分离出了CD133^+^肺癌细胞（LC^-^CD133^+^）和CD133^-^肺癌细胞（LCCD133^-^），并发现高表达*OCT4*基因的LC^-^CD133^+^有明显自我更新能力和无限增殖潜能，体内外放化疗实验均表明，LC^-^CD133^+^对放化疗抵御能力与*OCT4*基因表达有关，*OCT4*是参与调控胚胎干细胞自我更新和维持其全能性的最为重要的转录因子之一，同时也是体外建立诱导多功能干细胞（induced pluripotent stem cells, iPS）的关键基因，作者推断*OCT4*基因对于维持LC^-^CD133^+^的干细胞特性和耐化疗能力有重要意义。

Giangreco等^[7]^认为位于近端气道粘膜下腺体/内软骨环、神经上皮小体和终末细支气管/支气管肺泡交叉处的气道干细胞具有致癌特性，如高增殖能力、多向分化潜能和较长存活寿命。

美国学者^[8]^应用流式细胞仪从人类肺癌细胞系中分选出醛脱氢酶-1（aldehyde dehydrogenase 1, ALDH1）阳性的细胞，发现其具备诸多干细胞特征，并表达CD133；体内实验证明：ALDH1^+^细胞具备可形成与母代细胞异质性相同的肿瘤。此外，免疫组织化学分析303例临床标本发现ALDH1的表达与肺癌的分期和分级以及患者预后相关。因此作者大胆推测ALDH1可能是肺癌标记物和肺癌潜在的预后因子。

### 1.2 利用侧群细胞分选法分离肺癌干细胞

加拿大学者Ho等^[9]^应用Hoechst33342染色和荧光激活细胞分选方法，在6种肺癌细胞株（A549, H460, H23, HTB-58, H441, H2170）中分离出侧群细胞（side population cell, SP），并证实SP细胞具有自我更新、分化和高致瘤性的特征，并且发现其具备干细胞“永生”和处于静止状态的特征，高表达抗凋亡因子和端粒酶mRNA，而标志细胞增生的MCM7基因mRNA表达下调。因此他们认为肺癌的肿瘤干细胞主要富集在侧群细胞组分中，表达ABCG2等ATP结合盒（ATP binding cassette, ABC）转运蛋白。

Seo等^[10]^在研究A549人肺腺癌细胞株肿瘤干细胞*AKRICI/C2*、*TM4SFl*、*NROBI*基因表达时，他们利用SP细胞分选法成功将对数生长期的细胞分离为SP细胞与非SP细胞，并发现SP细胞*AKRICL/C2*、*TM4SFl*、*NROBI*基因表达上调并且具备肿瘤干细胞的生物学特征。

## 2 肺癌干细胞与肺癌形成

### 2.1 正常肺脏干细胞突变

从理论上来说，一个正常细胞转变为肿瘤细胞至少要发生4-7次突变，这需要几年或是几十年的时间。干细胞生存期长、分裂慢，最有可能长期受有害环境影响积聚突变向肿瘤干细胞转化。肺脏干细胞及前体细胞均有自我更新能力，有充分的时间来接受肿瘤形成所必须的遗传改变信息。当肺内正常干细胞自我更新的调节机制受损或肺干细胞积累了恶性变传递到肺的前体细胞，使其经历突变后重新获自我更新能力，都会导致肺干细胞无限增生，最终成肺癌^[11]^。因此，气管干细胞方面的研究也显得较为重要。

贾心善等^[12, 13]^应用5-氟尿嘧啶（5-Fluorouracil, 5-FU）对离体的人及大鼠气管造成损伤，建立了气管干细胞增殖分化动物模型^[14]^，对气管干细胞进行了定位研究。结果显示：5-FU损伤后，气管上皮细胞绝大部分脱落，可见少量间隔分布的类似裸核的细胞呈钉状位于基底膜上，PCNA染色阴性，证明为G_0_期细胞；去除5-FU后，正是通过这些干细胞增殖分化，修复了损伤的气管环。该实验室还证实，此G_0_期细胞能排除Hoechst33342染料，Bcrpl/ABCG2表达阳性^[15]^，Song等^[16]^发现此G_0_期细胞可表达胚胎干细胞标记物Oct3/4、Nanog和Sox2，进一步证明其为气管干细胞。此外，该损伤模型还可用于研究气管损伤修复过程中某些信号通路的开启情况及某些细胞因子的变化情况。

虽然干细胞和肿瘤干细胞密切相关，但应该注意到它们有着本质的不同^[17]^，例如肿瘤干细胞自我更新转导途径的负反馈机制已被破坏，倾向于累积复制的错误，而干细胞能够防止复制错误的发展。此外，干细胞增殖具有自稳定性，其数目在机体调控下保持恒定，而肿瘤细胞却无自稳定性的特点。

### 2.2 肺脏干细胞微环境调控异常

干细胞生长的微环境称之为干细胞“龛”（niche），它保持干细胞处于休眠状态；阻止其分化；保护肿瘤干细胞免于抗癌药物、放射线的攻击；通过信号传导通路调节着肿瘤干细胞的自我更新、分化、凋亡，影响着肿瘤干细胞的迁徙与转移。

研究^[18, 19]^发现肺癌的形成不仅与肺癌干细胞有关，肺脏局部微环境可能也参与肺癌形成，在不同解剖区域有不同的组织学表型。鼠类肺癌模型由近端气管至远端气管，大致分布的主要肺癌组织表型为：鳞癌、小细胞肺癌、支气管肺泡癌。这种在不同解剖区域有不同表型分布的现象提示：仅有少数散在分离的细胞及其微环境支持肺癌生长。*K-ras*基因变异小鼠模型实验也证实了这一观点：尽管整个小鼠气道细胞都发生了*K-ras*基因变异，但是仅位于支气管肺泡区域的细胞发生了腺瘤样增生。此外，肺腺癌是肺癌最常见的组织类型之一并且在细支气管和肺泡处多发。这表明：肺癌的形成不仅与致癌基因突变有关，还与不同细胞所处的微环境有关。利用小鼠鳞癌，腺癌，支气管肺泡癌模型确定的肿瘤起始位点与最新发现的气道干细胞所处的小生境位置似乎一致^[20]^。

### 2.3 肺脏干细胞信号转导机制异常

大量实验研究证明，正常干细胞和肿瘤干细胞具有相似的调节性信号转导通路，一旦正常干细胞自我更新的信号转导通路调节紊乱则会导致肿瘤增殖或形成肿瘤。目前已经清楚肿瘤干细胞的分化与自我更新受EGF/EGFR、PDGF/PDGFR、Notch和Wnt/β-catenin等通路的调控^[21]^。

#### 2.3.1 Wnt通路与肺脏干细胞

Wnt/β-catenin通路可以调控细胞周期，从而维持干细胞的分化和自我更新。王琳琳等应用5-FU大鼠气管上皮损伤模型，再现了气管干细胞原位增殖分化修复气管上皮的全过程，结果表明：Wnt-1时间依赖性表达与气管损伤修复进程相吻合，Wnt/β-catenin信号传导途径参与调控气管干细胞增殖分化全过程，修复早期促进气管干细胞的增殖，修复后期促进祖细胞向特定的细胞分化。张菁茹等^[22]^采用干细胞基因芯片比较受损气管上皮在恢复24 h及48 h与正常气管上皮基因表达的差异。24 h后差异基因8个上调，31个下调。48 h后5个上调，42个下调。差异基因表达主要集中在细胞周期调控、细胞连接、FGF、BMP分子、Notch、Wnt信号通路上。在肺干细胞中β-catenin主要通过减弱分化促进组织干细胞数量的增加，而不是促进干细胞直接增生，其在非小细胞肺癌干细胞中的作用也需要进一步研究^[23]^。

Mazieres等^[24]^证实在肺癌细胞信号转导通路中存在与上述干细胞相似的Wnt过度表达、非经典途径JNK通路激活和Wnt拮抗剂WIF-1的抑制和失活。Nakashima等研究证实，Wnt-1的过度表达会使肺癌发生演进。Kim等^[25]^应用转染后含有Wnt-1抑制因子基因的A549和H460细胞株，测定细胞增生，并将含有Wnt抑制因子的脂质体注射到移植瘤。结果表明Wnt-1抑制因子表达增加可以促进癌细胞的凋亡，使克隆形成率下降，并且重组的Wnt-1抑制因子蛋白能够抑制H460的增生，显著抑制肿瘤的生长。

#### 2.3.2 Notch通路与肺脏干细胞

在非小细胞肺癌干细胞的研究中，敲除小鼠基因的实验研究^[26]^表明Notch通路活化在肺的发育中起重要作用，并且Notch配体、受体和HES1的水平在非小细胞肺癌细胞株中表达升高，其与癌变的RAS信号关系密切^[27]^。应用γ-Secretase抑制剂能够抑制Notch2激活，在体内、外均可抑制肺癌的生长^[28]^。Ma等^[29]^采用大鼠损伤模型，分析了Notch信号分子Notch-3、Jagged-1和Hey1在气管干细胞增殖分化过程中的表达。实验证明，Hey-1的Notch信号在气管干细胞增殖分化过程中维持气管干细胞的未分化状态，促进气管干细胞的非分化性增殖。

就肺癌而言，其干细胞和肿瘤形成的关系正逐步揭晓。理论上，我们认为，上述三个条件在肺癌形成过程中均发挥了作用，但是其具体发生及调控机制目前尚不清楚，有待于进一步研究。

## 3 肺癌干细胞与临床治疗

### 3.1 肺癌干细胞可能与耐药性密切相关

目前大量的理论和实验均表明肿瘤干细胞可能与耐药性密切相关。与正常干细胞类似，生长缓慢和多处于休眠状态有助于肿瘤干细胞耐药性的产生。此外，肿瘤干细胞表面高表达多种ABC转运蛋白^[30]^，例如*ABCB1*基因编码P2糖蛋白（Pglycoprotein, Pgp）、*ABCG2/MXR*基因编码多药耐药相关蛋白1（multi-drug resistance related protein 1, MRP1）和乳腺癌耐药蛋白2（bcl-2），它们具备外排药物、毒物的能力。*ABCG2*、*ABCB1*或*ABCC1*基因敲除的小鼠对长春碱、异阿凡曼菌素、米托蒽醌等药物更加敏感^[31]^，提示ABC转运子可保护细胞不受化疗药物影响，也可能经过基因突变的积累，肿瘤干细胞把这种多药耐药机制遗传下来；或存在基因突变或分化异常导致复发的肿瘤出现了多药耐药性，这就可以解释为什么本来不具有耐药性的肿瘤在化疗后复发获得多药耐药性。

Sung等^[32]^也发现A549细胞株的侧群细胞亚群有*ABCG2*基因高表达，且*MDR1*和*ABCC2*基因高表达，并证明了其与肿瘤耐药性有关。

### 3.2 肺癌干细胞与肺癌复发

肺癌传统治疗方案中，放射疗法、化学药物疗法等被广泛应用于包括肺癌在内的各种恶性肿瘤，但是这些手段往往在治疗初期效果显著，但是维持时间短暂且伴有复发现象。放化疗手段杀伤的靶细胞为增殖活跃的肺癌细胞或定向前体细胞，而休眠的肺癌干细胞往往处于G_0_期，对射线和化学药物不敏感，可以抵御放化疗的杀伤，成为以后复发的根源^[33]^，这或许可以解释当前治疗恶性肿瘤失败的原因。因此针对肿瘤干细胞的治疗可能效果更持久，靶向去除肺癌干细胞将是治疗肺癌的新突破点。

### 3.3 清除肺癌干细胞的可能策略

研制ABC转运蛋白的阻断剂进而逆转肺癌干细胞的耐药性，充分发挥化疗药物的疗效。目前已批准进行临床研究的ABCG2抑制剂及其它ABCG2抗体的研究等^[34]^。

通过调控关键的信号通路来抑制肺癌干细胞的自我更新和诱导分化。可以通过小分子抑制剂如RNAi技术阻止这些信号通路的活化来治疗肿瘤。目前发现gamma蛋白分泌酶抑制剂，可以靶向Notch通路来抑制白血病和淋巴瘤细胞的生长^[35]^。

改善肿瘤干细胞的微环境，特别是血管生成。目前已经证实，抗血管内皮生长因子（vascular endothelial growth factor, VEGF）的单克隆抗体-贝伐珠单抗^[36]^（bevacizumab）对于结肠癌治疗有效。抗血管生成治疗已经应用在非小细胞肺癌的治疗中，并使患者的生存获益^[37]^。

直接靶定肺癌干细胞，根据特异性的表面分子标志识别肺癌干细胞，制备单克隆抗体，携带放射性物质或化疗药物，直接靶向放化疗，最终促使肺癌干细胞凋亡，使肿瘤失去生成新的肿瘤细胞的能力，争取达到根治的目的。通过肿瘤干细胞表面的分子抗原进行靶向杀伤。现已应用于临床的吉姆单抗（gemtuzumab）/奥佐米星（ozogamicin）是人源化的抗CD133单抗和细胞毒抗肿瘤抗生素刺孢霉素（calicheamicin）的偶联物，用于治疗复发的急性髓性白血病AML。最近发现抗CD44的单抗可以消除AML干细胞^[38]^。

## 4 总结

肺癌干细胞领域的研究刚刚起步，还有许多问题亟待解决。例如：探寻肺癌干细胞的具体生物学特性及其标记物、确定肿瘤干细胞与正常干细胞间的差别、明确其具体调控机制和信号转导通路等。但是，这一理论的提出及认定无疑将会是人类攻克肺癌的一大进步，可以解释肺癌的多种生物学行为，因此结合肿瘤干细胞学说，分析肺癌干细胞的生物学特性，进而阐明肺癌发生、发展、转移、耐药的机制，有助于肺癌的诊断、治疗和预后评估，有助于筛选靶向性杀伤肿瘤干细胞的药物，为临床提供新的治疗靶点，将会对肺癌的诊断、治疗及预防产生深远的影响。
